# Systemic CD4 cytotoxic T cells improve protection against PRRSV-1 transplacental infection

**DOI:** 10.3389/fimmu.2022.1020227

**Published:** 2023-01-17

**Authors:** Yanli Li, Ivan Díaz, Gerard Martín-Valls, Niklas Beyersdorf, Enric Mateu

**Affiliations:** ^1^ Departament de Sanitat i Anatomia Animals, Facultat de Veterinària, Universitat Autònoma de Barcelona (UAB), Cerdanyola del Vallès, Spain; ^2^ Centre de Recerca en Sanitat Animal, Institut de Recerca en Tecnologies Agroalimentáries (IRTA-CReSA), Universitat Autònoma de Barcelona (UAB), Cerdanyola del Vallès, Spain; ^3^ Institute for Virology and Immunobiology, University of Würzburg, Würzburg, Germany

**Keywords:** CTL, NKT, NK, PRRSV-1, transplacental infection

## Abstract

**Background:**

*Porcine reproductive and respiratory syndrome virus* (PRRSV) is one of the major swine pathogens causing reproductive failure in sows. Although modified-live virus (MLV) vaccines are available, only partial protection against heterologous strains is produced, thus vaccinated sows can be infected and cause transplacental infection. The immune effector mechanisms involved are largely unknown.

**Methods:**

The present study investigated the role of cytotoxic lymphocytes, including cytotoxic T cells (CTL), NKT, and NK cells, from blood in preventing PRRSV-1 transplacental infection in vaccinated primiparous sows (two doses vaccinated). Sows from a PRRSV-1 unstable farm were bled just before the last month of gestation (critical period for transplacental infection), then followed to determine whether sows delivered PRRSV-1-infected (n=8) or healthy (n=10) piglets. After that, functions of CTL, NKT, and NK cells in the two groups of sows were compared.

**Results:**

No difference was found through cell surface staining. But upon *in vitro* re-stimulation with the circulating field virus, sows that delivered healthy piglets displayed a higher frequency of virus-specific CD107a^+^ IFN-γ-producing T cells, which accumulated in the CD4^+^ compartment including CD4 single-positive (CD4 SP) and CD4/CD8α double-positive (CD4/CD8α DP) subsets. The same group of sows also harbored a higher proportion of CD107a^+^ TNF-α-producing T cells that predominantly accumulated in CD4/CD8α double-negative (CD4/CD8α DN) subset. Consistently, CD4 SP and CD4/CD8α DN T cells from sows delivering healthy piglets had a higher virus-specific proliferative response. Additionally, in sows that delivered PRRSV-1-infected piglets, a positive correlation of virus-specific IFN-γ response with average Ct values of umbilical cords of newborn piglets per litter was observed.

**Conclusion:**

Our data strongly suggest that CTL responses correlate with protection against PRRSV-1 transplacental infection, being executed by CD4 T cells (IFN-γ related) and/or CD4/CD8α DN T cells (TNF-α related).

## Introduction

*Porcine reproductive and respiratory syndrome virus* (PRRSV) is a positive-sense single-stranded RNA virus causing one of the costliest swine diseases worldwide. The virus causes reproductive failure in sows (*i.e.* late-term abortions, premature farrowing, stillbirths, and fetal mummification or weak-born piglets) and increased mortality of suckling piglets in farrowing units (1, 2). When weaned piglets are infected, the infection manifests as a respiratory disease often complicated by secondary agents (3).

Modified-live virus (MLV) vaccines are widely used for the control of PRRSV infections in endemic farms. For naïve breeders, vaccination usually starts before the first mating (1 or 2 doses), then recall vaccinations are administered periodically (often using a blanket protocol, every 3-4 months) to maintain the immunity. MLV vaccines are very effective to protect against homologous strains; however, only partial efficacy is shown during heterologous infections (4). Accordingly, the administration of PRRSV MLV vaccines helps to reduce the abortion rate but does not produce sterilizing immunity.

Vilalta et al. (5) showed that, in PRRSV-2 affected farms, birth of viremic piglets could be detected up to 23 weeks after the onset of an outbreak. It was reported that vertical transmission occurred particularly in younger sows, although they had been immunized and the inflow of new susceptible pigs was stopped. Our group found that the percentage of sows delivering infected piglets may reach 5-10% in PRRSV-1-vaccinated and -endemic farms in Spain, surprisingly, with no overt reproductive disease (unpublished data).

Previous studies suggested that homologous titers of neutralizing antibodies (NAb) ≥1:8-1:16 might protect against abortion or viremia in piglets (6, 7). Unfortunately, NAb against PRRSV usually have a narrow breadth and limited and unpredictable cross-neutralization efficacy (8). As a result, and given the high genetic/antigenic diversity of PRRSV, most natural exposures can be considered heterologous challenges. Protection in the absence of cross-neutralizing antibodies has been suggested to be the result of cell-mediated immune responses (4).

Cytotoxic lymphocytes play a key role in eliminating virus-infected cells through two mechanisms: 1) direct cytolytic activity mediated by releasing cytolytic proteins such as granzyme and perforin, or inducing death receptor-mediated apoptosis *i.e*. Fas ligand or TRAIL; 2) production of antiviral cytokines, most notably IFN-γ and TNF-α [reviewed by Barry and Bleackley (9)]. The classical cytotoxic cells are innate natural killer (NK) cells and the adaptive CD8 cytotoxic T cells (10). However, several studies have also uncovered the cytotoxic function of CD4 T cells that kill infected cells through mechanisms similar to NK and CD8 T cells (11, 12). CD4 cytotoxic lymphocytes (CD4 CTL) are MHC-II restricted, in contrast to classical CD8 CTL which are mediated by the MHC-I pathway (11, 12).

The role of cytotoxic lymphocytes in protection against transplacental infection of PRRSV is largely unknown. In this study, we followed a batch of primiparous sows and determined two groups, sows transmitting the infection to fetuses and sows that did not suffer transplacental infection. We then compared their CD4/CD8 CTL, NKT, and NK cells in blood through a range of assays, including cell surface staining, intracellular staining of IFN-γ and TNF-α, and proliferative responses to the field circulating virus. The results showed that upon *in vitro* re-stimulation, sows delivering healthy piglets had a higher frequency of CD107a^+^ IFN-γ-producing T cells, which accumulated within the CD4^+^ compartment including CD4 single-positive (CD4 SP) and CD4/CD8α double-positive (CD4/CD8α DP) subsets. Also, the same group of sows harbored a higher proportion of CD107a^+^ TNF-α-producing T cells that mainly accumulated in the CD4/CD8α double-negative (CD4/CD8α DN) subset. The proliferative response of CD4 SP and CD4/CD8α DN subsets to the virus was also higher in sows that delivered healthy piglets. Our data strongly suggest that CTL responses are correlated with the protection against PRRSV-1 transplacental infection, being executed by CD4 T cells or CD4/CD8α DN cells.

## Materials and methods

### Farm and sampling of gilts

**Figure 1:**
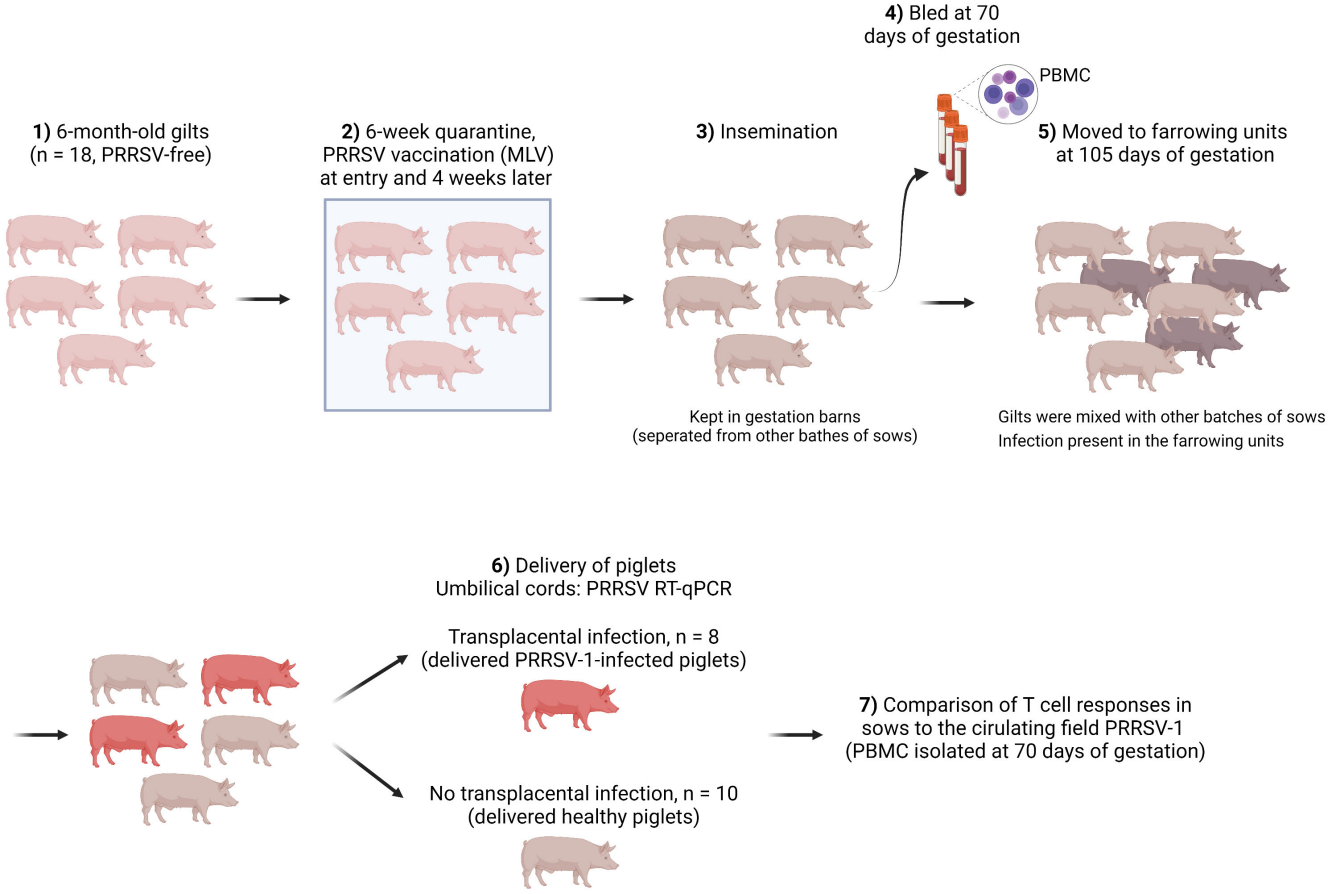
Schematic of gilt management, sampling, and determination of transplacental infection.

Gilts that were followed in this study belonged to a 500-sow farm with farrowing batches every two weeks. No reproductive failure above normal values was observed when the farm was selected. **Figure 1** shows the schematic of gilt management in the farm. Specifically, gilts were purchased at 6-6.5 months of age from a certified PRRSV-free source. Then gilts were allocated to an isolated quarantine where they stayed for at least 6 weeks. All gilts were vaccinated with a PRRS MLV vaccine (Porcilis^®^ PRRS, MSD Animal Health, Salamanca, Spain) upon entry into the quarantine unit and were revaccinated 4 weeks later with the same vaccine.

After quarantine, gilts were mated (insemination with certified PRRSV-free semen) and kept in the mating units until gestation was confirmed (day 35 of gestation). Afterwards, gilts were re-located to a gestation area and stayed until day 105 of gestation. Finally, gilts were moved to the farrowing units. The farm housed pregnant sows with static groups. In other words, animals in the same batch were allocated to the same pen, where they could have contact with each other but physically separated from other batches of sows. Therefore, the involved gilts only contacted with gilts in the same batch before being moved to the farrowing units (day 105), where they interacted with sows from other batches (until the expected delivery date, days ~114-115 of gestation). Gilts were bled on day 70 of gestation, which was 35 days after moving to the gestation units and 35 days before transferring to the farrowing units.

No clinical signs were observed before gilts were moved to the farrowing units. Upon delivery, umbilical cords from the newborns were collected for PRRSV diagnosis by RT-qPCR. Weaners (6-7 weeks of age) from other batches were also tested. Both were diagnosed positive for PRRSV-1. The isolated virus was a strain with increased virulence corresponding to a cluster recently reported (Genbank accession number ON571708). Whole-genome sequencing revealed that the virus was derived from the highly virulent Italian strain PR40 (13) with at least three additional recombination events with other PRRSV-1 isolates (14). In the affected farm, 10% of sows suffered from reproductive failures, *i.e.* late abortions, premature farrowing, fetal death, and the birth of congenitally infected piglets, since introduction of the new virulent strain. The impact of this virus resulted in a decrease in the global production of the farm, from 32 to 27 piglets/sow/year. In addition, mortality reached 20% in nurseries as a consequence of the outbreak, mostly caused by secondary bacterial infections.

### Isolation of peripheral blood mononuclear cells (PBMC)

PBMC were separated by density-gradient centrifugation within 4 h after sampling. Blood was diluted 1:2 with Hank’s balanced salt solution (Fisher Scientific, Spain) and layered onto Histopaque 1.077 (Sigma-Aldrich, Spain) in SepMate tubes (STEMCELL Technologies, France) before centrifugation (400 × g, 30 min) at room temperature. The cellular interface was retrieved, and red blood cells were lysed in 0.15M NH4Cl. Between each step, washing 3× with Dulbecco’s PBS (DPBS, Fisher Scientific, Spain) was done. Cells were counted and the viability was determined by trypan blue staining in a Neubauer chamber. Finally, cells were frozen in Cryostor CS10 (Sigma-Aldrich, Spain) at approximately 20 × 10^6^/vial (1 ml CS10) and stored in liquid nitrogen until used.

### Determination of vertical transmission: sows delivering PRRSV-1-infected or PRRSV-1-free piglets

Umbilical cords of newborn piglets were collected to assess the incidence of transplacental infection. Briefly, 3-5 cm of umbilical cords were aseptically collected in sterile DPBS (Fisher Scientific, Spain) immediately after the birth of piglets. Once received in the laboratory, umbilical cords were sliced in sterile PBS. Within each litter, suspensions of umbilical cords were pooled every two piglets. RNA was then extracted using MagMAX Pathogen RNA/DNA Kit (Thermo Fisher Scientific, Spain). Transplacental infection was determined when at least one pool was positive in PRRSV-1 RT-qPCR (VetMAX PRRSV EU & NA 2.0 Kit, Thermo Fisher Scientific, Spain).

### Isolation and propagation of the virus

Sera from infected sows and piglets were used to isolate the circulating virus in porcine alveolar macrophages (AM). AM were seeded in 24-well plates (1 × 10^6^/well, Sigma-Aldrich, Spain) and allowed to attach overnight. Then, cells were inoculated with the RT-qPCR positive sera (250 μl/well) that had been diluted 1:5 with MEM containing 100 units/ml penicillin, 100 μg/ml streptomycin, and 50 μg/ml gentamicin (all from Fisher Scientific, Spain). At 1.5 h post-infection, the inoculum was washed away and fresh medium containing 7.5% fetal bovine serum (FBS, Sigma-Aldrich, Spain) was added. The isolated virus, designated as JA2, was then titrated on AM with titer determined by the immunofluorescence assay using an anti-PRRSV-1 nucleocapsid (N) antibody (clone 1C5H, Ingenasa, Spain). Further propagation of JA2 was performed in cell culture flasks (25 cm^2^, Sigma-Aldrich, Spain) with 10 × 10^6^ macrophages seeded. The 4^th^ passage with titer 10^6.5^ TCID_50_/ml was used in the present study. The full genome of the virus was sequenced using RNAseq Illumina Technology as reported before (15).

### Flow cytometry analysis of CTL NKT NK phenotype

Cryopreserved PBMC were thawed, rested overnight at 37°C, then counted for use in the following experiments. The viability of PBMC from sows that delivered infected and healthy piglets was respectively 91.8 ± 3.1% and 92.9 ± 3.5% after thawing, and 85.8 ± 14.3% and 85.0 ± 12.8% after rest. Cell number mentioned below means viable cells.

Five-color staining (live/dead and CD3/CD4/CD8α/CD16) was performed to define CTL, NKT, and NK cells. PBMC (500,000) were initially stained with LIVE/DEAD Near-IR dead cell stain (1 μl/ml, Thermo Fisher Scientific, Spain) on ice for 30 min. Then, cells were labeled with an anti-CD16 antibody (clone FcG7, Bio-Rad, Spain) followed by anti-mouse IgG1 conjugated to BV421 (BioLegend, Spain), and finally with a cocktail of CD3-PE (clone BB23-8E6-8C8, BD Biosciences), CD4-FITC (clone 74-12-4, BD Biosciences, Spain), and CD8α-AF647 (clone 76-2-11, BD Biosciences, Spain). To further characterize NK cells, CD4-FITC was replaced with NKp46-AF488 (clone VIV-KM1, Bio-Rad). At least 100,000 cells were acquired using a MACSQuant Analyzer 10 (Miltenyi Biotec, Bergisch Gladbach, Germany).

In all assays of the present study, cell surface staining was performed in DPBS containing 5% FBS and 5% horse serum (Sigma-Aldrich, Spain) for 30 min on ice. Between each step, cells were washed twice with DPBS (2% FBS). Antibodies involved were titrated before use. Their specificities were affirmed by comparing with the matched isotype antibodies. Live lymphocytes were defined by forward/side scatter, height/area forward scatter, and live/dead stain. Single staining of each fluorochrome was prepared for compensation. Positive events were gated using fluorescence minus one (FMO) controls. Analysis was performed using FCS Express 7 (*de novo* Software, Glendale, CA, United States).

### Cell Trace Violet proliferation assay

PBMC were labeled with 5 μM CellTrace Violet (Thermo Fisher Scientific, Spain) at 37°C for 20 min in the dark. After washing 3×, 250,000 cells/well were added to the 96-well round-bottom plates (Sigma-Aldrich, Spain). Then, cells were cultured for 3 days with PRRSV-1 JA2 (multiplicity of infection (MOI) 0.1) in quadruplicates (final volume 200 μl) in complete RPMI supplemented with 10% FBS, 100 units/ml penicillin, and 100 μg/ml streptomycin. Mock (complete RPMI) and PHA (10 µg/ml) stimulation were used as negative and positive controls, respectively. Proliferation for 3 days was determined according to a kinetic study (1, 3, and 5 days), where the viability of lymphocytes on day 3 was around 45% and 5-10% of CD3^+^ T cells had proliferated upon JA2 stimulation, whereas after 5 days, < 20% of viable lymphocytes were remained (data not shown). At the end of incubation, cells from four wells were mixed and stained with Near-IR dead cell stain followed by biotinylated anti-CD16 antibody (clone FcG7, BD Biosciences) that were revealed by streptavidin PerCP-Cy5.5 (BD Biosciences, Spain). A mixture of CD3-PE, CD4-FITC, and CD8α-AF647 was finally added. At least 250,000 events were acquired. The proportion of cells that had proliferated in the culture was calculated with the following formula: (sum of cells in all generations)/(total number of cells of the subset) × 100%.

### IFN-γ and TNF-α intracellular cytokine staining assay

After overnight rest, PBMC (250,000) were stimulated with PRRSV-1 JA2 (MOI 5.0) or mock (complete RPMI) stimulated in the presence of CD107a-FITC (2 μg/ml, clone 4E9/11, Bio-Rad, Spain) and co-stimulatory monoclonal antibody anti-CD28 [1 μg/ml, clone 3D11 (16)] at a final volume of 200 μl. Cultures were produced in octuplicates and incubated for 8 h with brefeldin A and monensin added for the final 4 h. After incubation, cells were harvested by washing once with DPBS, and then every four replicas were mixed to reach 1 × 10^6^ cells for staining in 96-well V-bottomed plates. Live cells were identified by using the fixable Near-IR staining as described above. Afterwards, cells were stained with a mixture of CD3-Pacific Blue (clone PPT3, Bio-Rad), CD4-PE-Cy7 (clone 74-12-4, BD Biosciences, Spain), and CD8α-PE (clone 76-2-11, BD Biosciences, Spain). Next, cells were fixed and permeabilized (BD Cyfix/Cytoperm, BD Biosciences, Spain), and finally intracellularly stained with an anti-IFN-γ-AF647 antibody (clone P2G10, BD Biosciences, Spain) at 4°C for 30 min. For TNF-α staining, CD8α-PE was substituted by CD8α-AF647 and an antibody anti-TNF-α (clone 103302, R&D Systems, Spain) conjugated to LYNX Rapid RPE (Bio-Rad, Spain) was used. A minimum of 50 positive events were acquired for either IFN-γ^+^ or TNF-α^+^ in CD3^+^ cells.

### IgG ELISPOT assay

PRRSV-specific IgG secreting cells (IgG-SC) were measured utilizing a commercial ELISPOT kit (Porcine IgG ELISPOT BASIC, Mabtech, Nacka Strand, Sweden) according to the manufacturer’s instructions. Briefly, nitrocellulose-bottomed plates (MultiScreen-HA plates, Merck, Spain) were coated overnight at 4°C with PRRSV-1 JA2 at MOI 0.1 in DPBS. The MOI was chosen according to a preliminary dose-response test. PBMC were added at 500,000 cells/well (in 200 µl complete RPMI) and left stimulated overnight before plate development. All tests were run in triplicates. The number of PRRSV-specific IgG-SC were calculated by subtracting counts of spot-forming units (SFU) in unstimulated wells from counts in virus-stimulated wells. PRRSV-specific IgG-SC were expressed as SFU/10^6^ cells.

### Statistical analysis

All data were analyzed using the GraphPad Prism 9.3 software package (GraphPad Software, La Jolla, CA, United States). The method of statistical tests applied to each data set is indicated in the figure legend.

## Results

Examination of umbilical cords from the delivered piglets by RT-qPCR divided sows into two groups: one (n = 8) delivering PRRSV-1-infected piglets and the other (n = 10) giving birth to PRRSV-1-free piglets (also mentioned as healthy piglets).

### Surface profiling of potential CTL, NKT, and NK cells did not detect differences between sows that delivered PRRSV-1-infected and healthy piglets

**Figure 2:**
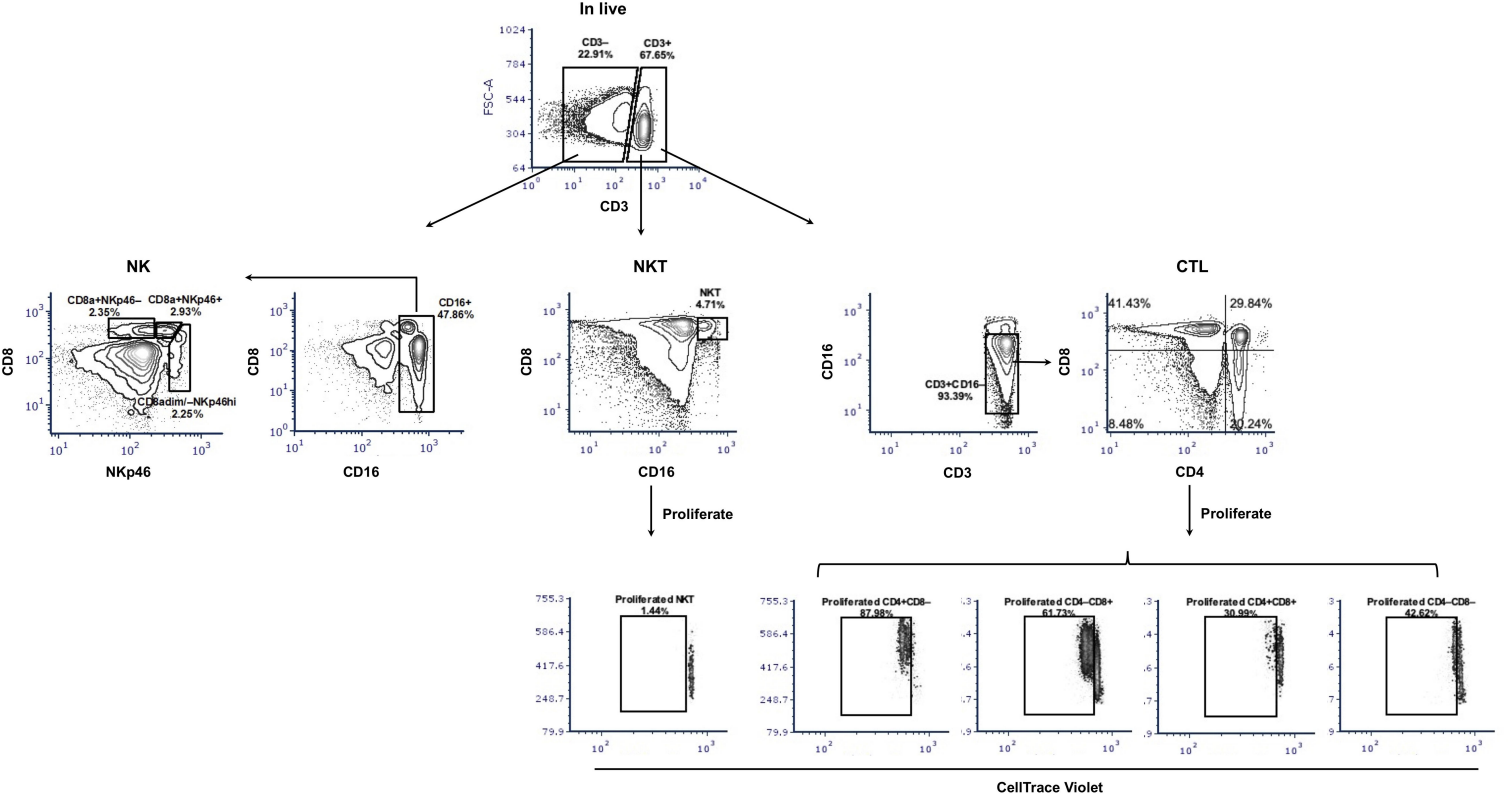
Gating hierarchy of surface staining to identify potential CTL, NKT, and NK cells and their proliferation. The gating hierarchy is shown as contour plots. For phenotyping, a five-color staining scheme was performed: live/dead Near-IR/CD3/CD4/CD8α/CD16. After removal of cell debris and gating on singlets, live cells were divided based on the expression of CD3. Within CD3-positive cells, NKT cells were defined as CD16^+^CD8α^+^ and potential CTL as CD4/CD8α subsets within CD3^+^CD16^–^ cells. In the CD3-negative population and after gating CD16^+^ cells, three subsets of NK cells were shown: CD8a^+^NKp46^–^, CD8a^+^NKp46^+^, and CD8a^dim/–^NKp46^hi^. For proliferation assays, CellTrace Violet-labeled PBMC were stimulated with PRRSV-1 JA2 (MOI 0.1) for 3 days, then collected and stained for live/dead Near-IR/CD3/CD4/CD8α/CD16. The proliferated CTL and NKT were defined as CellTrace Violet^dim^ within each subset. Stimulation with medium only or PHA was used as negative and positive controls, respectively.

We firstly examined the proportion of potential CTL, NKT, and NK cells in PBMC obtained from blood of sows at the 10^th^ week of gestation through cell surface staining. Potential CTLs were considered to belong to the CD3^+^CD16^–^ subset with further classification based on CD4 and CD8α expression (gating hierarchy shown in **Figure 2**). Potential NKT cells were defined as CD3^+^CD16^+^CD8α^+^ (**Figure 2**). Statistically, no difference in the proportion of potential CTL or NKT was shown between sows with and without transplacental infection (**Table 1**), but a slightly higher frequency of CD4 single-positive T cells (CD4 SP, CD4^+^CD8a^–^) and a slightly lower frequency of CD4/CD8a double-negative T cells (CD4/CD8a DN) were seen in sows delivering healthy piglets in the whole PBMC and also in T cells (CD3^+^) (**Table 1**).

**Table:** Table 1 Percentage of T cell subsets, NKT, NK cells, and B cells.

		Sows _infected piglets	Sows _healthy piglets	Sows _infected piglets	Sows _healthy piglets	Sows _infected piglets	Sows _healthy piglets
		**In whole PBMC**		**In CD3^+^**		**In CD3^–^**	
**T cells**		64.0 ± 6.5	63.3 ± 6.3	–	–	–	–
	**CD4 SP**	7.2 ± 3.5	9.8 ± 4.0	12.9 ± 6.8	16.7 ± 5.5	–	–
	**CD8a SP**	21.0 ± 5.1	20.5 ± 3.0	40.8 ± 6.2	41.4 ± 3.3	–	–
	**CD4/CD8a DP**	17.0 ± 8.2	16.5 ± 4.2	29.1 ± 12.2	28.5 ± 6.4	–	–
	**CD4/CD8a DN**	9.6 ± 3.6	7.4 ± 3.3	17.2 ± 6.9	13.4 ± 6.1	–	–
**NKT**		3.8 ± 1.4	4.0 ± 1.3	5.9 ± 2.0	6.4 ± 1.7	–	–
**NK**		1.4 ± 0.7	1.2 ± 0.3	–	–	7.7 ± 4.2	6.1 ± 1.8
	**CD8a^+^NKp46^–^**	0.5 ± 0.3	0.5 ± 0.2	–	–	2.8 ± 1.3	2.4 ± 1.4
	**CD8a^+^NKp46^+^**	0.5 ± 0.4	0.4 ± 0.2	–	–	2.9 ± 2.1	1.8 ± 0.8
	**CD8a^dim/-^NKp46^hi^**	0.4 ± 0.2	0.4 ± 0.2	–	–	2.0 ± 1.3	1.8 ± 1.0
**B cells**		19.0 ± 6.5	17.5 ± 5.4	–	–	–	–

No significant difference was found between the two groups of sows for all subsets.

Whole PBMC: Acquired events with debris and dead cells excluded;

T cells: CD3^+^;

CD4/CD8a subsets: All subsets were gated within CD3^+^CD16^–^. CD4^+^CD8α^–^ (CD4 single-positive, CD4 SP), CD4^–^CD8α^+^ (CD8a single-positive, CD8 SP), CD4^+^CD8α^+^ (CD4/CD8a double-positive, CD4/CD8a DP), CD4^–^CD8α^–^ (CD4/CD8a double-negative, CD4/CD8a DN) subsets within CD3^+^CD16^–^ population.

NKT: CD3^+^CD16^+^CD8α^+^;

NK: CD8a^+^NKp46^–^, CD8a^+^NKp46^+^, and CD8a^dim/–^NKp46^hi^ subsets within CD3^–^CD16^+^ population;

B cells: CD21^+^.

For NK cells, three subsets were defined by the expression of CD8a and NKp46 within CD3^–^CD16^+^ cells (**Figure 2**). They were two classical CD8a-positive subsets CD8a^+^NKp46^–^ and CD8a^+^ NKp46^+^, and a third subset assumed to have a higher cytotoxic capacity CD8a^dim/–^NKp46^hi^ (17). Sows delivering infected piglets showed a higher frequency of CD8a^+^NKp46^+^ in CD3^–^ cells (on average 2.9 vs 1.8%) although the difference was not significant and was narrowed in the whole PBMC (0.5 vs 0.4%) (**Table 1**).

### Sows that did not suffer transplacental infection harbored a higher frequency of degranulated IFN-γ- and TNF-α-producing T cells that accumulated in CD4 T cells and CD4/CD8α DN T cells respectively

**Figure 3:**
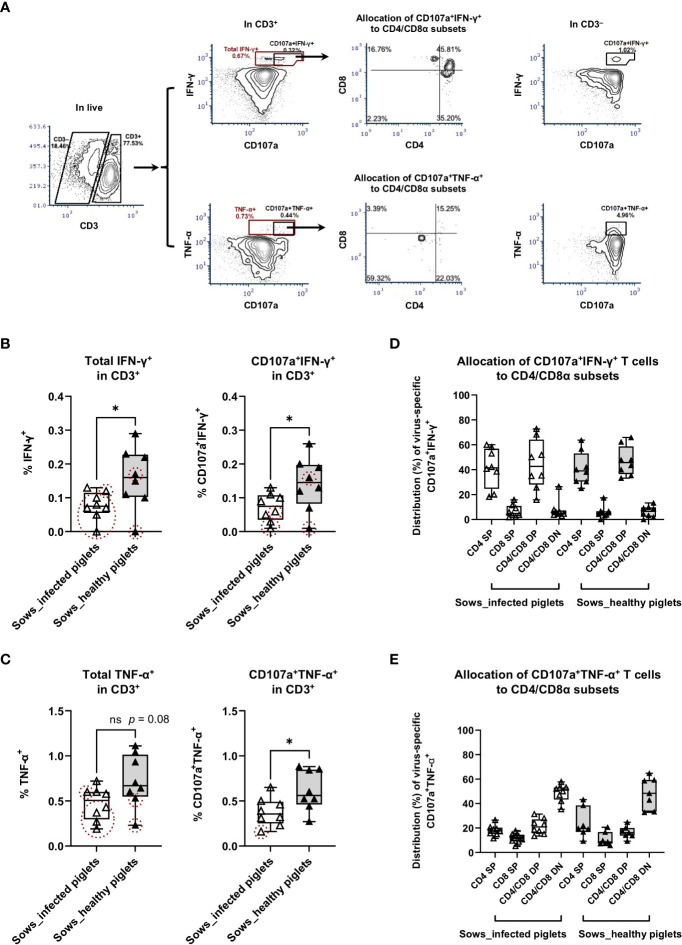
Virus-specific IFN-γ and TNF-α responses as measured by intracellular cytokine staining assays. PBMC from sows with or without the occurrence of transplacental infection were stimulated with PRRSV-1 JA2 strain *in vitro* for 8 h in the presence of anti-CD107a and anti-CD28 antibodies and with brefeldin A and monensin added for the final 4 h. After that, cells were stained for live/dead Near-IR, CD3/CD4/CD8α, and intracellular IFN-γ or TNF-α. **(A)** Gating hierarchy to identify virus-specific IFN-γ- and TNF-α-producing T cells with cell degranulation: exclusion of debris → singlets → live cells → CD3^+^/CD3^–^ → total IFN-γ^+^ or CD107a^+^IFN-γ^+^, total TNF-α^+^ or CD107a^+^TNF-α^+^ → CD4/CD8α; **(B)** Virus-specific IFN-γ responses and **(C)** virus-specific TNF-α responses in T cells (CD3^+^). The graph shows the proportion of total IFN-γ-producing and CD107a^+^ IFN-γ-producing T cells, total TNF-α-producing and CD107a^+^ TNF-α-producing T cells in PRRSV-1 JA2-stimulated cultures. Sixteen sows (8 per group) were included. Each symbol, representing one sow, was the mean percentage of two replicas with background (cultures in the absence of PRRSV-1 JA2 stimulation) subtracted. Empty boxes (empty triangles) represent sows delivering infected fetuses; solid boxes (solid triangles) represent sows delivering healthy piglets. Symbols marked by dashed circles (in red) mean the frequency of IFN-γ- or CD107a^+^ IFN-γ-producing, or TNF-α- or CD107a^+^ TNF-α-producing T cells in JA2-stimulated cells was < 2-fold that of mock-stimulated cells; Allocation of virus-specific **(D)** CD107a^+^ IFN-γ-producing T cells and **(E)** CD107a^+^ TNF-α-producing T cells to CD4/CD8α subsets. Statistical significance was measured by the Mann–Whitney nonparametric test. *p < 0.05.

To investigate whether there was a functional divergence of cytotoxic lymphocytes between sows delivering PRRSV-1-free and -infected piglets, we performed an intracellular cytokine (IFN-γ and TNF-α) staining combined with degranulation marker CD107a and cell surface CD3, CD4, and CD8α. For sows delivering healthy piglets, two were not included in this analysis due to low accessible cell number. Antigen-specific T cells were defined as IFN-γ- or TNF-α-producing T cells, while potential CTL were defined as degranulated antigen-specific T cells (18), namely cells with positive labeling for CD107a and IFN-γ or TNF-α (**Figure 3A**). NKT cells were not included in this analysis due to the limitation of fluorochrome combinations.

For total IFN-γ-producing T cells upon PRRSV-1 JA2 stimulation, sows delivering healthy piglets showed a significantly higher frequency than their counterparts delivering infected piglets (*p* < 0.05, **Figure 3B**). A similar tendency was also seen for TNF-α-producing T cells (*p* = 0.08, **Figure 3C**). Notably, in sows that delivered infected piglets, 7/8 and 5/8 of them (marked as dashed red circles) did not reach the positivity criteria (> 2-fold of mock-stimulated cells) for frequencies of IFN-γ^+^ (**Figure 3B**) and TNF-α^+^ (**Figure 3C**) T cells, respectively. By contrast in the group that did not suffer transplacental infection, only 2/8 of sows were off the positivity criteria. This suggests that sows suffering transplacental infection harbored much lower or even negligible virus-specific T cell. Further combination with degranulation marker CD107a improved the selection of virus-specific T cells, where only 3/8 and 1/8 of sows delivering infected piglets were off the positivity criteria for CD107a^+^IFN-γ^+^ and CD107a^+^TNF-α^+^ T cells, respectively. No difference was seen in the total proportion of CD107a^+^ T cells between two groups of sows (**Supplementary Figure 1**).

CTL response with regards to IFN-γ production (CD107a^+^ IFN-γ-producing T cells) to *in vitro* PRRSV-1 JA2 (MOI 5.0) stimulation is shown in **Figure 3B**. Although CD107a^+^ IFN-γ-producing T cells were detected in both groups, sows that delivered healthy piglets showed a significantly higher level (0.15 ± 0.07%) compared to their counterparts suffering transplacental infection (0.07 ± 0.04%) (*p* < 0.05) (**Figure 3B**). This is by contrast with in CD3^–^ cells where no difference was shown between the two groups (**Supplementary Figure 2A**). Further phenotyping revealed that CD107a^+^ IFN-γ-producing T cells mostly accumulated in the CD4^+^ T compartment, including CD4 SP and CD4/CD8α DP (**Figure 3D**). The same pattern was seen in both groups of sows (without transplacental infection: 40.8 ± 13.3% in CD4 SP and 44.3 ± 20.0% in CD4/CD8α DP; with transplacental infection 41.5 ± 15.9% and 47.0 ± 11.8% respectively; non-significant) (**Figure 3D**).

For CTL response with regards to TNF-α production (CD107a^+^ TNF-α-producing T cells), sows that delivered healthy piglets also showed a higher frequency, on average 0.63 ± 0.24%, contrasting with 0.38 ± 0.16% in sows suffering transplacental infection (*p* < 0.05) (**Figure 3C**). Responsive cells were predominantly accumulated in the CD4/CD8α DN subset (48.6 ± 12.9 vs 48.8 ± 7.2% of sows delivering healthy and infected piglets, respectively) (**Figure 3E**). Within CD3^–^ population, a higher proportion of CD107a^+^ TNF-α-producing cells was also detected in sows that gave birth to healthy piglets (**Supplementary Figure 2B**). But more than 90% of those cells were CD4/CD8α DN, indicating they were not NK cells.

**Figure 4:**
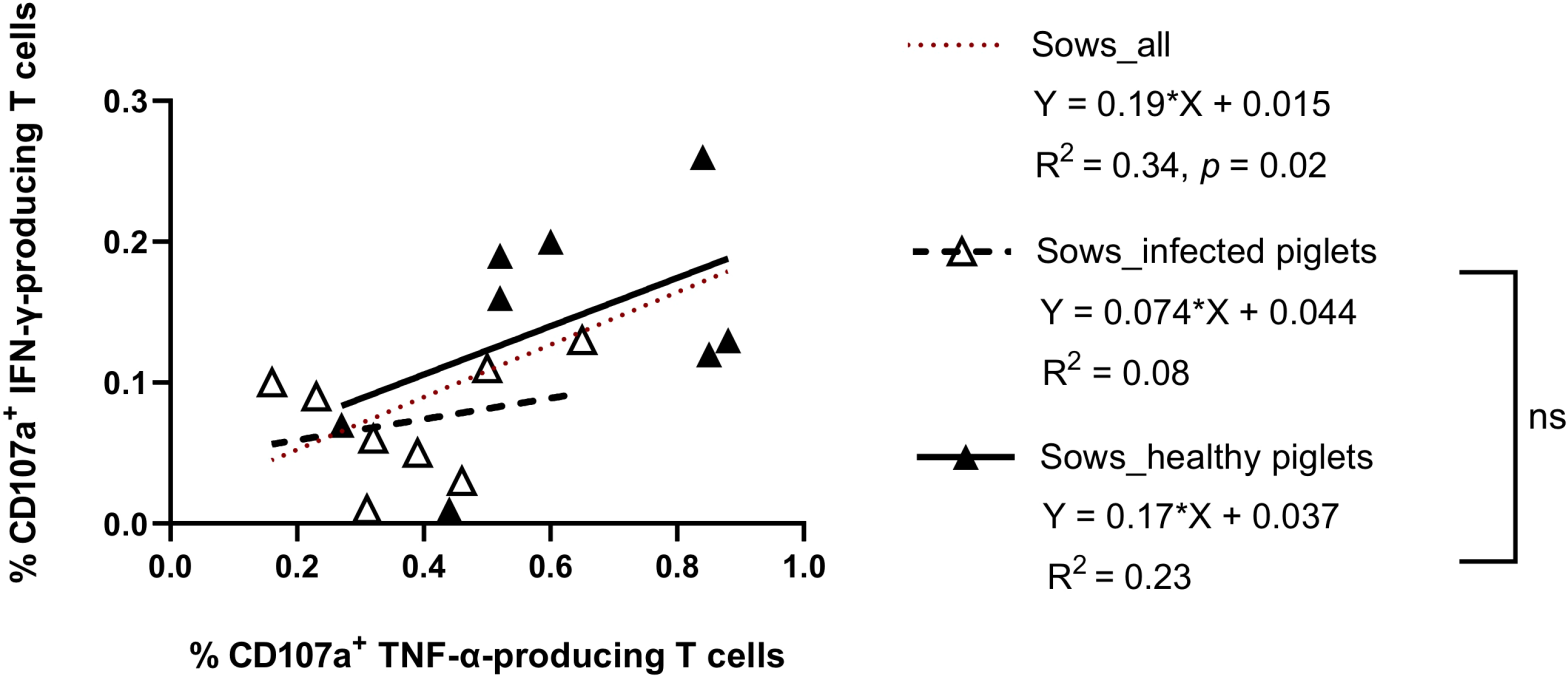
Scatter plots showing the relationship between CD107a^+^ IFN-γ- and CD107a^+^ TNF-α-producing T cells in sows delivering PRRSV-1 JA2-infected and healthy piglets. Dashed line (in red), linear regression line of the relationship between CD107a^+^ IFN-γ-producing T cells and CD107a^+^ TNF-α-producing T cells considering all animals., Empty triangle, sows delivering infected piglets; solid triangle, sows delivering healthy piglets. Dashed line (in black), linear regression of sows delivering JA2-infected piglets; solid line (in black), linear regression of sows delivering healthy piglets. Slopes for the regression lines of healthy and infected animals were not significantly different, ns: not significant.

On the individual level (considering all examined sows), the frequency of virus-specific CTL responses showed a significant correlation between IFN-γ- and TNF-α production (IFN-γ = 0.19 + 0.015 TNF-α; R^2^ = 0.34, *p* = 0.02) (**Figure 4**). Comparing the two groups, there was a tendency towards higher responses for both cytokines in sows giving birth to healthy piglets (slope regression 0.13 vs 0.10, **Figure 4**), although the difference was not statistically significant. This means the relationship between IFN-γ and TNF-α responses was similar in both groups, but sows that did not suffer transplacental infection harbored a higher strength of response.

### Infection status of newborn piglets correlated with the frequency of IFN-γ-producing T cells in sows

**Figure 5:**
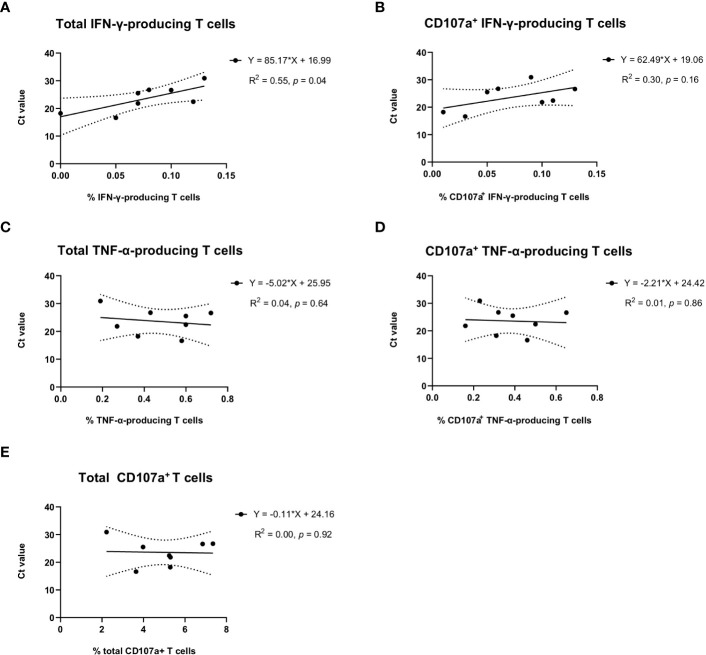
Correlation of virus-specific IFN-γ, TNF-α, or CD107a T cells in sows with the average Ct values of umbilical cords of newborn piglets per litter. Average Ct values of umbilical cords from the same litter of newborn piglets were used to indicate the infection status of newborn piglets. Solid lines, linear regression of the correlation between Ct values of newborn piglets and PRRSV-1 JA2-specific **(A)** total IFN-γ-producing or **(B)** CD107a^+^ IFN-γ-producing T cells, **(C)** total TNF-α-producing or **(D)** CD107a^+^ TNF-α-producing T cells, and **(E)** total degranulated (CD107a^+^) T cells detected in each sow. Each solid circle represents one sow that delivered infected piglets. The value was the mean percentage of two replicas with background (cultures in the absence of PRRSV-1 JA2 stimulation) subtracted. Dashed lines, 95% confidence intervals; R^2^, fit goodness; *p* values, slope significance.

In the group of sows delivering JA2-infected piglets, we observed that average Ct values of newborn piglets from the same littler showed a significant correlation with the frequency of total IFN-γ-producing T cells (R^2^ = 0.55, *p* = 0.04) (**Figure 5A**) but not with CD107a^+^ IFN-γ-producing T cells (R^2^ = 0.30, *p* = 0.16) (**Figure 5B**) in the blood of sows. This suggests a higher level of virus-specific IFN-γ-producing T cells obtained from MLV vaccination may contribute to alleviating infection severity of fetuses when transplacental infection happens.

No correlation was observed with virus-specific TNF-α response (**Figures 5C, D**) or overall T cell degranulation (**Figure 5E**).

### CD4 SP and CD4/CD8α DN T cells proliferated to a higher extent in sows that delivered healthy piglets

**Figure 6:**
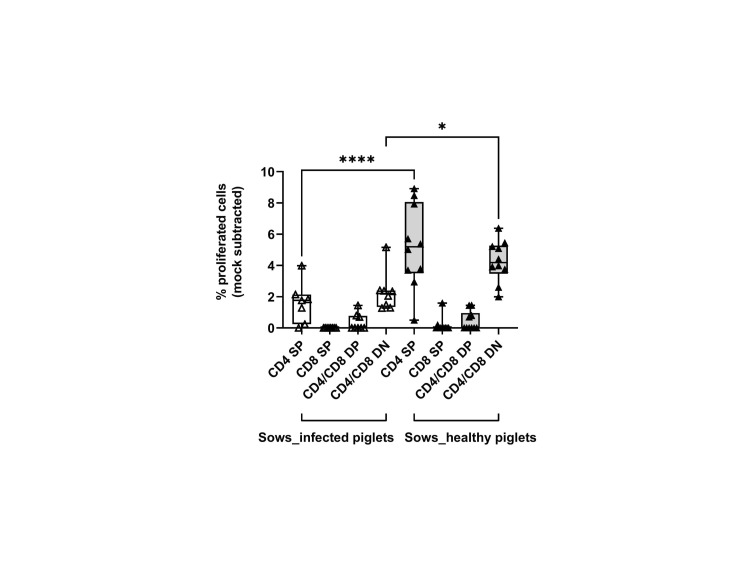
Proliferative responses of T cells from sows that delivered infected or healthy piglets to PRRSV-1 JA2 stimulation. PBMC labeled with CellTrace Violet were stimulated *in vitro* with the circulating field virus at MOI 0.1. After 3 days, a five-color staining, live/dead Near-IR/CD3/CD4/CD8α/CD16, was performed. In each T cell subset (CD4 SP, CD8a SP, CD4/CD8a DP, and CD4/CD8a DN), the proportion of CellTrace Violet^dim^ cells was defined as the ratio of proliferation. Mock-stimulated cells were subtracted from the virus-stimulated cultures. Empty boxes, sows delivering JA2-infected piglets; grey-filled boxes, sows delivering healthy piglets. Statistical significance was measured by the Mann–Whitney nonparametric test, *p < 0.05, ****p < 0.0001.

We then measured the proliferative response of lymphocytes from each sow (gating hierarchy shown in **Figure 1**). Upon *in vitro* re-stimulation with PRRSV-1 JA2 (MOI 0.1), CD4 SP and CD4/CD8α DN T cells proliferated to a higher extent in sows that delivered healthy piglets than their counterparts delivering JA2-infected fetuses (5.2% vs 1.6% for CD4 SP, and 4.3% vs 2.3% for CD4/CD8α DN, *p* < 0.05) (**Figure 6**). Of note, the proliferated cells (CellTrace Violet^dim^) were mostly Near-IR^+^, namely dead cells (data not shown). This indicates a short half-life of the virus-specific cells in *in vitro* re-stimulated cultures, possibly caused by exhaustion or activation-induced cell death (AICD) (19). By contrast, CD8α SP and CD4/CD8α DP as well as NKT and NK cells rarely proliferated in either group of sows (data not shown).

### A higher frequency of virus-specific IgG-SC was detected in sows delivering healthy piglets in *ex vivo* IgG ELISPOT

**Figure 7:**
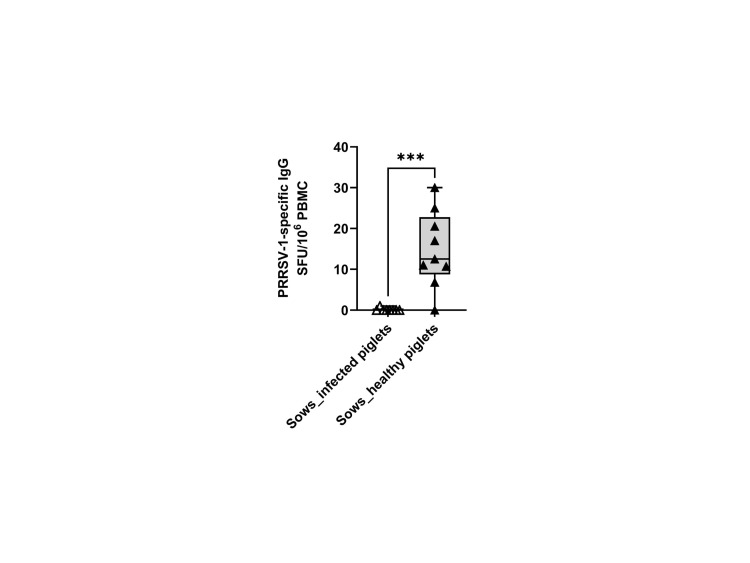
Frequencies of virus-specific IgG-secreting cells (IgG-SC) in sows that delivered infected and healthy piglets. PBMCs (500,000 cells) were stimulated overnight *in vitro* with the circulating field virus JA2 at MOI 0.1 followed by IgG ELISPOT development. Statistical significance was measured by the Mann–Whitney nonparametric test, ***p < 0.001.

We also examined the frequency of memory B cells through *ex vivo* IgG ELISPOT. The results showed that only sows delivering healthy piglets showed apparent counts in the IgG ELISPOT (14.8 ± 9.3 vs 0.1 ± 0.4 SFU/10^6^ cells) (**Figure 7**). Since the ELISPOT was performed without expansion of Ag-specific B-cell clones, the SFU enumeration would correspond mostly to plasma cells or long-lived plasma cells (20).

## Discussion

Transplacental infection of PRRSV has profound implications in the field: the crucial mechanism for maintaining the infection in herds and the leading cause of increased mortality of suckling piglets [reviewed by Pileri and Mateu (21)]. Transplacental infection takes place in late gestation, after 80 days of pregnancy (22). Although the precise causes are not fully known, it has been suggested that CD163^+^ and sialoadhesin^+^ macrophages (PRRSV susceptible cells) are enriched in the placenta at 80 days after gestation. This, together with changes in the endometrial vascularization, may explain why transplacental infection happens only at the end of gestation (22).

Although MLV vaccines help reduce the impact of PRRSV-associated reproductive problems, it is not uncommon to have vaccinated sows transmitting the virus to fetuses after exposure to a heterologous strain during gestation. The underlying mechanisms by which vaccinated sows remain susceptible or become resistant to transplacental heterologous infection are largely unknown. NAb were proposed to afford protection against abortion in the homologous challenge model (6). Unfortunately, NAb usually have a narrow neutralization breadth and lack broadly neutralizing capacity (8). Hence in our study, protection from transplacental infection in some sows was unlikely to arise from NAb, especially when the circulating strain was highly virulent and genetically distant from the vaccine strain (>18% difference, at the whole genome level, in our study). In an unpublished study from our group, sera from gilts vaccinated with the same protocol were unable to neutralize any of the heterologous PRRSV-1 strains tested (n = 5). The scarce capacity of vaccine-induced NAb for heterologous infection suggests an essential role of cell-mediated immunity.

The primary goal of this study was to explore the role of virus-specific cytotoxic lymphocytes including CTL, NKT, NK cells in preventing PRRSV-1 transplacental infection. To this end, we followed a cohort of gilts during gestation and determined two groups of sows: the one delivering healthy piglets and the other delivering infected piglets. Using PBMC collected just before the last month of gestation, we compared the frequency of virus-specific cytotoxic responses between two groups of sows. Gilts were kept in isolation during the immunization phase with no contact with older sows before late gestation. This guaranteed the immunity against PRRSV-1 at the moment of sampling was gained only from vaccination. A lateral introduction of a new strain at the end of the gestation allowed us to investigate the efficacy of immunity developed in sows against a heterologous virus. Considering fast transmission to sows in the farrowing units, high rate of infection, and the fact that mortalities surpassed 20% in both maternity and nursery phases, the new strain presented a worst-case scenario, a virulent easy-to-transmit PRRSV-1 strain. With all gilts sharing the same air space in the farrowing units, it is unlikely that gilts not deliver infected piglets were not exposed to the virus. But it is not assured whether the exposed gilts did not develop viremia or antigen-specific T cells blocked the transplacental infection. An experimental model will be needed to clarify this point.

Initially, we did cell surface staining to examine overall immune profile of different cell subsets, including potential CTL, NKT, and NK cells, at 70 days of gestation. No differences were found between the two groups of sows, suggesting the outcome of exposure did not affect the overall frequencies of immune cells in peripheral blood. But the use of distribution measurements in PBMC may differ from the result of absolute counts in the whole blood. That should be considered for future study.

We then performed the intracellular staining of IFN-γ and TNF-α combined with the degranulation marker CD107a and cell surface CD3, CD4, and CD8α. CD107a^+^ IFN-γ-producing T cells were detected in both groups of sows as expected after administration of the vaccine. Not surprisingly, sows that delivered PRRSV-1-free piglets showed a higher frequency. However, instead of the classical CD8 CTL, CD107a^+^ IFN-γ-producing T cells were mainly restricted to CD4^+^ T cells, including both CD4/CD8α DP and CD4 SP subsets. Correspondingly, CD4 SP displayed a higher proliferative response to the field virus in sows delivering healthy piglets, whereas proliferation of CD8α SP was negligible. This agreed with a previous study, where the killing of autologous PRRSV-infected macrophages was carried out by CD4 but not CD8 T cells (23). CD4/CD8 DP with CTL features were also detected by Meier et al. (24) and Cao et al. (25), who additionally proposed the contribution by CD8 SP. Nevertheless, in Cao’s study, CD107a was rarely detected on CD8 SP (25), suggesting they were non-cytotoxic effectors. Also, Cao’s and Meier’s studies used pigs infected by homologous strains, in contrast to sows exposed to a heterologous highly virulent strain in our study.

In the past decade, CD4 CTL have been identified in human and mouse models, not only during viral responses but also in anti-tumor and autoimmune responses [reviewed by Takeuchi and Saito (12)]. Like CD8 CTL, CD4 CTL contribute to viral clearance *via* secreting antiviral cytokines and, in some conditions, secreting granzyme B and perforin to kill the target cells in an MHC class II-restricted fashion. CD4 CTL have been mostly reported in chronic viral infections such as human cytomegalovirus and human immunodeficiency virus (26–28), but also in acute infections such as influenza when CD8 responses were impaired (29). A cytotoxic-Th1 cluster with high avidity and clonally expanded capability was also detected in COVID-19 patients who developed milder symptoms (30).

The role of CD4 CTL in swine infectious diseases is largely unknown, although some clues could be traced from literatures. Lohse et al. (31) showed that depletion of CD8 cells did not exacerbate PRRSV infection. In another study (32), cytotoxic CD8^+^ T cells could not be detected in the first 56 days after inoculation and thereafter only in some animals at low intensity. These evidences suggest that classical CD8 CTL may not be the major actor in host defense against PRRSV. Rather, a higher level of CD4 CTL-skewed memory response gained from vaccination would be crucial. In our study, a pivotal role of CD4 CTL in protecting against transplacental infection was distinguished. Whereas both groups of sows produced CD4 CTL after PRRSV-1 MLV prime-boost, sows harboring a higher frequency of CD4 CTL did not suffer transplacental infection. Therefore, we hypothesize that higher levels of PRRSV-specific CD4 CTL are crucial for sows to clear heterologous infection and to protect from transplacental infection. In the study of Kick et al. (33), CD4 T cells were also proved to be the major responder to PRRSV-2 in blood, but CD8 T cells responses were assumed to prevail in the lung. Therefore, the predominant T cell type combating PRRSV infection is likely to be different between blood and tissues, indicating different roles of circulating and tissue-resident T cells. Also, more studies are required to resolve whether discrepancies exist in response to PRRSV-1 and PRRSV-2 or to strains with different virulence. Kick et al. (33, 34) showed that PRRSV-2-vaccinated animals only responded to the highly pathogenic strain. Moreover, the role of memory CTL response (central/effector/stem-like memory) would be crucial to uncover the development of host immunity against PRRSV.

Two factors are assumed to drive the development of CD4 CTL, long-term exposure to viral antigens (35) and deficient or impaired CD8 CTL response (29). Coincidentally, a common feature of PRRSV infection is viral persistence in lymph nodes for an extended period after clearance of viremia. As demonstrated by Bierk et al. (36), transmission from infected sows may happen 86 days after the onset of infection. It is still unknown whether CD8 CTL could be transiently induced and execute cross-cytotoxicity. In the future, it would be valuable to characterize the dynamic development of CD4/CD8 effector and memory CTL elicited by PRRSV MLV vaccines, and the efficacy to protect homologous and heterologous infections. It is also critical to evaluate the presence of CD4 CTL in tissues at different phases as CD4 CTL are thought to reside mainly in tissues (12).

Besides CD107a^+^ IFN-γ-producing T cells, the second correlate of protection observed in our study was CD107a^+^ TNF-α-producing T cells. More than half of these cells accumulated in the CD4/CD8α DN subset, which contains mainly TCRγδ^+^ T cells. As has been shown in several species, CD4/CD8 DN T cells could engage in innate and adaptive immune responses against different pathogens, acting as CTL secreting TNF-α under certain conditions [reviewed by (37)]. In the case of PRRSV, Ladinig et al. (38) reported that higher levels of γδ T cells were correlated with lower viral loads in an experimental infection of gilts, although in that study fetal mortality was not correlated. The role of γδ T cells in the development of immunity against PRRSV was also proposed by Olin et al. and Kick et al. (33, 34).

Notably, IFN-γ and TNF-α CTL responses were significantly correlated in our study, suggesting both types of cells may coordinate to protect sows from transplacental infection. Since the regression slopes were similar between two groups of sows, sows with higher susceptibility to transplacental infection likely represent a subpopulation of low responders with regards to the examined parameters.

Besides protecting sows from transplacental infection, virus-specific IFN-γ-producing T cells were likely to reduce the viral load reaching fetuses when transplacental infection happened. In the group of sows delivering infected piglets, the frequency of virus-specific IFN-γ- but not TNF-α-producing T cells positively correlated with the Ct values of newborn piglets. The mechanism may relate to the reduction of virus produced in placenta, resulting then in less virus crossing placental barrier or largely delay of the vertical transmission. In this case, no correlation with virus-specific TNF-α-producing T cells was detected. But given that only eight animals per group were examined, caution is advised in the interpretation of the results due to only factors with high impact could be detected in the present design.

Although protection was very unlikely to arise from NA as mentioned above, it is worth mentioning that sows that did not transmit the virus to their offspring had a higher level of memory B cells as detected by IgG ELISPOT. Since IgG ELISPOT was performed using virus-coated plates without expansion of pre-formed B cell clones, the result suggested an enhanced B cell memory response to viral envelope proteins in the protected sows. Non-neutralizing antibodies (nNAb), induced by MLV vaccination or natural infection, may not prevent PRRSV infection or even enhance the severity of the disease. However, their functional capabilities with regards to other mechanisms in clearing PRRSV-infected cells are still largely unknown. For human viral pathogens, multifunctional nNAb have long been recognized and studied in terms of antibody-dependent cell-mediated cytotoxicity (ADCC), antibody-dependent cell-mediated phagocytosis (ADCP), complement-mediated cytotoxicity, and spatially blocking proteins for viral replication (39). Examples of nNAb acting synergistically with T cells have also been reported (40, 41). This probably coincides with the observation in our study that enhanced virus-specific T cells along with a higher frequency of IgG-SC conferred heterosubtypic immunity to sows to protect transplacental infection. It would be interesting to perform a further assessment of the role of nNAb in PRRSV. The results of the present study suggest that protected sows were high responders compared to sows that suffered transplacental infection. Our group previously proved that increased IFN-γ responses and higher levels of NA in high responders to PRRSV-1 vaccination were related to the host genetic background (42).

Taken together, our study demonstrated that differences between sows delivering healthy and PRRSV-1-infected piglets were mainly quantitative, not qualitative based on the fact that PRRSV-1-specific T cell responses were detectable in both groups of sows. The results highlighted the potential role of CD4 CTL and CD4/CD8α DN cells in protection against transplacental infection. Because of the high genetic distance between the vaccine and field-circulating strain (>18%), cross-reactive T cell epitopes are assumed to be involved. It would be a breakthrough if cross-reactive and promiscuous T-cell epitopes, eliciting the IFN-γ producing CD4 CTL and TNFα-secreting γδ T cells, could be determined in the future. Exploration of newer adjuvants promoting such responses is also demanded, as differences in sows delivering PRRSV-1-free and -infected piglets are mainly quantitative. Additionally, a promising vaccine should consider the host’s genetic background.

## Data availability statement

The original contributions presented in the study are included in the article/**Supplementary Material**. Further inquiries can be directed to the corresponding authors.

## Ethics statement

The animal study was reviewed and approved by Committee for Human and Animal Experimentation of the Universitat Autònoma de Barcelona n° 5709.

## Author contributions

YL and EM conceived the study, designed experiments, analyzed and interpreted data, wrote and edited the manuscript; YL produced the virus, performed RT-qPCR and all flow cytometry assays; ID isolated PBMC and performed IgG ELISPOT; GM-V contacted farms, sampled gilts, collected clinical data, performed RT-qPCR, and isolated the virus; NB produced anti-CD28 monoclonal antibody. EM supervised the work. All authors have approved the final version of the manuscript.
