# Supplementary material for: Systemic CD4 cytotoxic T cells improve protection against PRRSV-1 transplacental infection
Source: Front Immunol. 2023 Jan 17;13:1020227. doi: 10.3389/fimmu.2022.1020227 (PMC9928156; doi:10.3389/fimmu.2022.1020227)
Supplement: Supplementary file 1 [file DataSheet_1.docx]

Supplementary Material

## Supplementary Figures

**
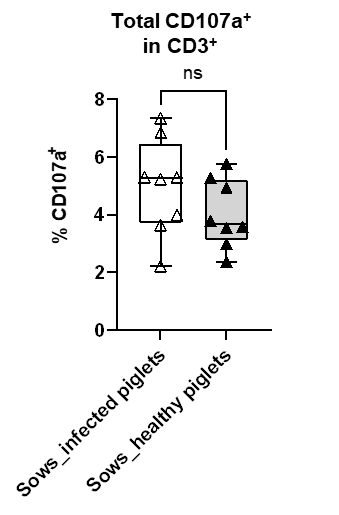
**

**Supplementary Figure 1.** **Total proportion of CD107a^+^ T cells after stimulation with PRRSV-1 JA2 strain *in vitro* for 8 h in the presence of anti-CD107a and anti-CD28 antibodies.**

**
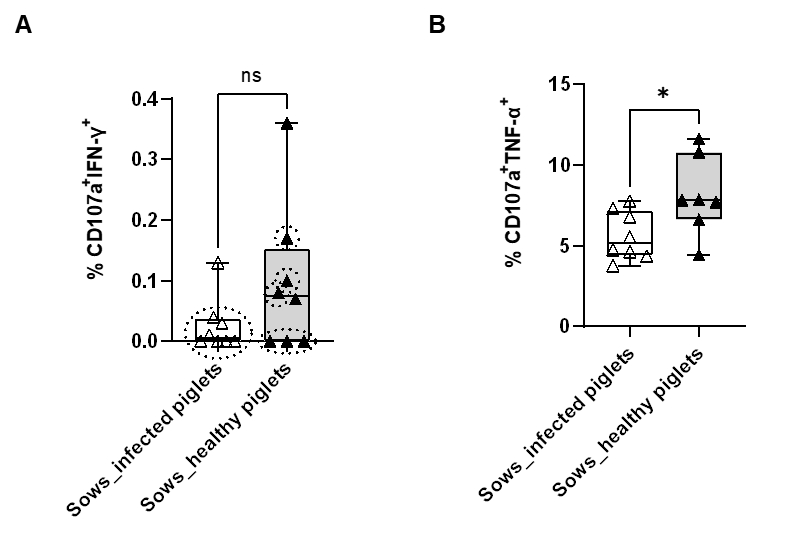
**

**Supplementary Figure 2.** **Virus-specific IFN-γ and TNF-α responses in CD3^–^ cells measured by intracellular cytokine staining assays.** The graph shows the proportion of (A) CD107a^+^ IFN-γ-producing and (B) CD107a^+^ TNF-α-producing CD3^–^ cells in PRRSV-1 JA2 stimulated cultures with background subtracted (cultures in the absence of JA2). Empty boxes (empty triangles) represent sows delivering infected fetuses; grey-filled solid boxes (solid triangles) represent sows delivering healthy piglets. Symbols marked by dashed circles mean, in sows, the frequency of CD107a^+^ IFN-γ-producing T cells in JA2-stimulated cells was < 2-fold that of non-stimulated cells. Statistical significance was measured by the Mann–Whitney nonparametric test; ns, not significant, **p* < 0.05.

**
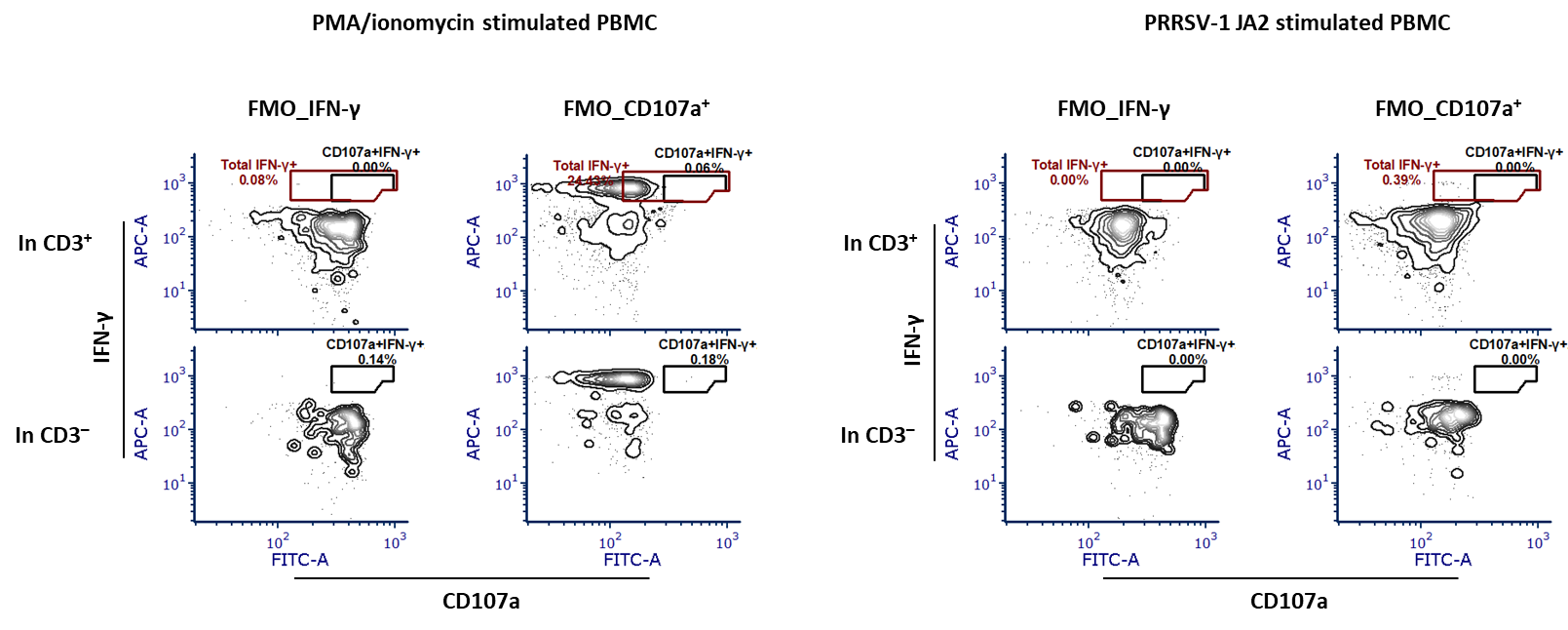
**

**Supplementary Figure 3.** **FMO controls of IFN-γ and CD107a staining.** PBMC from one of the examined sows that were stimulated with PMA/ionomycin (positive control of cytokine production and cell degranulation) or PRRSV-1 JA2 were used to prepare FMO controls. Details of cell stimulation and staining were described in “Materials and Methods”.
